# Comparative Neuroplasticity in Frontal‐ and Lateral‐Eyed Mammals With Induced‐Binocular Vision Dysfunction: Insights From Monocular Deprivation Models

**DOI:** 10.1111/ejn.70179

**Published:** 2025-07-09

**Authors:** Fábio Leite do Amaral Júnior, Thalyta Alves Rodrigues, Nonata Lúcia Trévia da Silva, Izabela Negrão Almeida Diniz, Luciana Negrão Almeida de Morais, Daniel Guerreiro Diniz, Dora Brites, Daniel Clive Anthony, Cristovam Wanderley Picanço Diniz

**Affiliations:** ^1^ Laboratório de Investigações em Neurodegeneração e Infecção Universidade Federal do Pará, Instituto de Ciências Biológicas, Hospital Universitário João de Barros Barreto Belém Pará Brazil; ^2^ Instituto Evandro Chagas Seção de Hepatologia, Laboratório de Microscopia Eletrônica Belém Pará Brazil; ^3^ Universidade Federal do Pará, Núcleo de Pesquisas em Oncologia, HUJBB Belém Pará Brazil; ^4^ Research Institute for Medicines (iMed.ULisboa) Universidade de Lisboa, Faculdade de Farmácia Lisboa Portugal; ^5^ Departamento de Ciências Farmacêuticas e do Medicamento Universidade de Lisboa Lisbon Portugal; ^6^ Department of Pharmacology, Laboratory of Experimental Neuropathology University of Oxford Oxford UK

**Keywords:** amblyopia, extracellular matrix, glial cells, monocular deprivation, visual cortex

## Abstract

Visual cortical plasticity during early postnatal life is profoundly shaped by species‐specific ocular anatomy and ecological demands. This review synthesizes comparative evidence on how monocular deprivation (MD)—a classical model of amblyopia—affects visual system development in frontal‐ versus lateral‐eyed mammals. Frontal‐eyed species, including cats and primates, exhibit extensive binocular field overlaps and columnar architecture in the primary visual cortex (V1), making them highly susceptible to MD‐induced shifts in ocular dominance and synaptic remodeling. In contrast, lateral‐eyed species such as rodents and ungulates possess limited binocular overlaps and lack well‐defined ocular dominance columns yet still demonstrate significant MD‐induced plasticity involving callosal reorganization, glial activation, and extracellular matrix remodeling. We examine shared and divergent cellular mechanisms underpinning these responses, including the role of parvalbumin‐expressing interneurons, perineuronal nets, and neuromodulators like BDNF and NRG1. Rodent models support the notion that even in the absence of classical columnar organization, lateral‐eyed species can undergo region‐specific structural remodeling in V1 following MD. These distinctions underscore how binocular integration circuits are fine‐tuned through extended critical periods in frontal‐eyed species, whereas plasticity in lateral‐eyed species is more diffusely distributed. The integration of cross‐species data revealed conserved principles of visual cortical plasticity and identified mechanisms potentially targetable for amblyopia therapy. Understanding the ecological and anatomical context of plasticity allows for a more accurate interpretation of animal models and supports the development of precision strategies for visual rehabilitation. This comparative framework expands the scope of amblyopia research and offers new avenues for translational interventions.

## Graphical abstract

This visual comparison illustrates the disparity in binocular visual fields between lateral‐eyed (A, mouse) and frontal‐eyed (B, cat) mammals. Mice exhibit a narrow binocular field of approximately 35°, while cats display a much broader overlap of about 98°, highlighting fundamental differences in sensory integration and visual cortex organization across species.

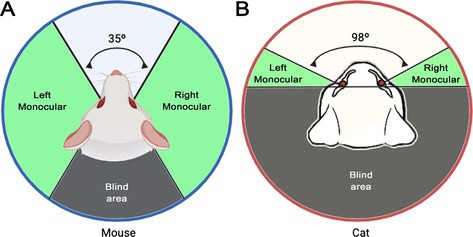

## 1 Introduction

Neuroplasticity refers to the capacity of the central nervous system to reorganize structurally and functionally in response to intrinsic and extrinsic factors, including sensory input, environmental influences, and injury (Bavelier et al. 2010; Kolb and Whishaw 1998). In the visual system, this adaptive capacity is particularly evident during defined postnatal critical periods—windows of heightened synaptic plasticity during which visual experience sculpts neuronal connectivity and receptive field properties (Hensch 2005a; Hubel and Wiesel 1963). This is exemplified by amblyopia, in which altered sensory input during the critical period leads to persistent deficits in visual perception and changes in cortical organization.

Amblyopia is a developmental disorder of spatial vision caused by abnormal visual experience—such as strabismus, anisometropia, or form deprivation—early in life (Holmes and Clarke 2006). It is associated with reduced visual acuity and contrast sensitivity in one eye and represents a failure of the binocular visual system to develop normally (Levi 2013). Experimental models of amblyopia frequently employ monocular deprivation (MD), wherein one eye is occluded during the critical period to simulate unbalanced input. This leads to profound changes in ocular dominance, synaptic strength, and structural features within the primary visual cortex (Gordon and Stryker 1996; Wiesel and Hubel 1965).

This review focuses on how MD impacts visual system plasticity in mammals with distinct ocular anatomies: frontal‐eyed and lateral‐eyed species. Frontal‐eyed animals such as cats and non‐human primates possess a high degree of binocular field overlap and exhibit well‐defined ocular dominance columns (ODCs) in their primary visual cortex (LeVay et al. 1980). These species rely heavily on binocular integration for depth perception and stereopsis, making them particularly vulnerable to MD‐induced disruptions during early development (Crair et al. 1998). In contrast, lateral‐eyed species, including rodents, rabbits, and ungulates, have laterally placed eyes resulting in minimal binocular overlap and dominance of monocular visual processing. Their visual cortices lack clear ODCs and instead rely more extensively on interhemispheric callosal projections and subcortical pathways (Laing et al. 2015).

Understanding these interspecies differences is essential as they influence both the magnitude and the nature of plastic responses to MD. For example, while MD in cats leads to a rapid shift in ocular dominance and synaptic pruning in the deprived‐eye columns, MD in rodents produces more diffuse and regionally restricted alterations, often involving callosal reorganization and glial activation (Laing et al. 2015; Pietrasanta et al. 2012; Restani and Caleo 2016). Furthermore, the neurochemical and cellular substrates underlying critical period closures, such as the maturation of parvalbumin (PV)‐positive interneurons, formation of perineuronal nets (PNNs), and expression of neuromodulatory signals—such as NRG1/ErbB4—may differ in their expression patterns and functional significance between these two visual system architectures (Berardi et al. 2004; Gu et al. 2016; Pizzorusso et al. 2002; Wen et al. 2018).

This review aims to identify convergent and divergent cellular mechanisms that govern visual circuit remodeling by comparing MD‐induced plasticity across frontal‐ and lateral‐eyed mammals. These findings, contextualized within a comparative framework, may inform the design of targeted therapeutic interventions for amblyopia and related neurodevelopmental disorders. In doing so, we underscore the need to interpret animal model data considering species‐specific neuroanatomy and visual ecology, fostering translational relevance and scientific rigor.

## 2 Experimental and Clinical Foundations of Amblyopia

Amblyopia remains a leading cause of monocular vision loss in children and young adults worldwide (Holmes and Clarke 2006; Simons 2005). The condition is classically categorized into three main types based on etiology: 1. strabismic amblyopia, caused by misalignment of the visual axes; 2. anisometropic amblyopia, resulting from unequal refractive error; and 3. deprivation amblyopia, associated with obstructed vision due to conditions such as congenital cataract (Birch 2013). All three forms share a common neurophysiological basis, that is, an imbalance in patterned visual input to the developing cortex that disrupts normal binocular integration and results in synaptic competition favoring the dominant eye (Daw 1998; Levi 2013).

The experimental foundations of amblyopia trace back to the seminal work of Hubel and Wiesel, who demonstrated that MD during a sensitive developmental window in kittens leads to dramatic alterations in cortical response properties, including a shift in ocular dominance and atrophy of deprived‐eye inputs in the primary visual cortex (Hubel and Wiesel 1963; Hubel and Wiesel 1965). These findings provided direct evidence that visual experience drives functional and structural plasticity in cortical circuits and that deprivation‐induced changes are irreversible beyond the critical period (Berardi et al. 2000). Subsequent studies expanded this paradigm to monkeys, ferrets, and rodents, revealing conserved and divergent aspects of deprivation‐induced plasticity (Espinosa and Stryker 2012; Gordon and Stryker 1996; Issa et al. 1999; LeVay et al. 1980).

Clinically, amblyopia is typically diagnosed based on a reduction in the best‐corrected visual acuity in one eye, unaccounted for by structural abnormalities (Holmes 2014; Holmes and Clarke 2006). The severity of vision loss varies depending on the timing, duration, and type of deprivation. For instance, deprivation amblyopia often produces more profound deficits than anisometropic forms, and earlier onset correlates with poorer prognosis (Epelbaum et al. 1993; Keech and Kutschke 1995). Neuroimaging and electrophysiological studies in humans have confirmed cortical hypoactivation in the amblyopic eye and disruptions in binocular fusion (Anderson and Swettenham 2006; Hess et al. 2010). These changes are often accompanied by broader cognitive and motor impairments, including delays in reading acquisition, visuomotor coordination, and spatial attention (Ho and Giaschi 2009; Kelly et al. 2015).

From a translational perspective, animal models have been indispensable in elucidating the cellular and molecular mechanisms of amblyopia. In particular, studies in cats and monkeys have shown that MD reduces dendritic spine density, disrupts excitatory‐inhibitory balance, and leads to the reorganization of thalamocortical input patterns (Casagrande and Joseph 1980; Hofer et al. 2009; Movshon 1976; Shatz and Stryker 1978). Rodent models, while lacking the laminar complexity and columnar architecture of the primate visual cortex, have been instrumental in identifying key regulators of critical period plasticity, such as brain‐derived neurotrophic factor (BDNF), GABAergic interneurons, and activity‐dependent transcription factors (Hensch 2005a; Huang et al. 1999; Levelt and Hübener 2012).

It is important to recognize that most experimental studies have been conducted in frontal‐eyed species with highly developed binocular systems. Lateral‐eyed animals, although historically underutilized in amblyopia research, provide a contrasting framework in which binocular integration plays a more limited role. In these species, the consequences of MD are more subtle and localized, often manifesting as alterations in interhemispheric callosal connectivity or the properties of monocular zones (Pietrasanta et al. 2012; Restani and Caleo 2016). However, recent data suggest that lateral‐eyed species can still exhibit significant glial (L. L. Wang et al. 2022) and extracellular matrix remodeling (Oray et al. 2004) in response to early visual deprivation, indicating that amblyopia‐related plasticity is not exclusive to species with ODCs.

Together, clinical observations and experimental studies across species support a model in which early visual experience is essential for proper development of binocular circuitry and that disruption of this input leads to a cascade of neuroplastic changes with long‐term perceptual consequences. By integrating findings from frontal‐ and lateral‐eyed species, we can develop a more comprehensive understanding of the biological underpinnings of amblyopia and better tailor interventions to the specific visual ecologies of affected individuals.

## 3 Comparative Effects of Amblyopia: Frontal‐ Versus Lateral‐Eyed Mammals

The comparative analysis of how MD impacts frontal‐eyed and lateral‐eyed mammals provides a unique framework for understanding visual cortical plasticity's evolution and specialization. Eye positioning fundamentally influences the organization and function of visual systems. Frontal‐eyed mammals—such as cats, ferrets, and primates—have substantial binocular field overlap and rely on stereoscopic vision for depth perception, which is mediated through complex integration in cortical circuits like ODCs in V1 (Horton and Hocking 1996; Hubel and Wiesel 1968; LeVay et al. 1980; Mitchell and Sengpiel 2009; Wiesel and Hubel 1963a, 1963b). In contrast, lateral‐eyed species—including rodents, rabbits, and many ungulates—have visual fields that minimally overlap and are optimized for panoramic vision, depending more on monocular and subcortical processing and less on binocular fusion (Heesy 2004; Hughes 1971; Laing et al. 2015; Priebe and McGee 2014).

In frontal‐eyed animals, the effects of MD are robust and well‐characterized. Studies in kittens by Wiesel and Hubel (1963a, 1963b) revealed that MD leads to a profound shift in ocular dominance, such that the open eye gains synaptic territory in V1 at the expense of the deprived eye (Wiesel and Hubel 1963a, 1963b). These changes are evident anatomically—through shrinkage of geniculocortical terminals—and functionally—through loss of visual responsiveness to the deprived eye (Antonini and Stryker 1993; Shatz and Stryker 1978). Similar findings have been reported in macaques, where MD causes long‐lasting deficits in binocular visual function, including stereoacuity and binocular summation (Crawford et al. 1989; Harwerth et al. 1989; Kiorpes 2019; Movshon et al. 1987). ODCs are prominent features in the primary visual cortex of the mentioned frontal‐eyed species (Katz and Crowley 2002). These columns serve as a structural framework for understanding the cortical reorganization that occurs following MD, making these species invaluable models for mechanistic studies of visual plasticity (Crair et al. 1998; Issa et al. 1999; Yacoub et al. 2007).

Lateral‐eyed species such as rodents, though lacking classical ODCs, show consistent and measurable plastic changes in response to MD (Takahata 2024). In mice, short periods of MD during the critical period led to shifts in visual evoked potential (VEP) amplitude and intrinsic signal imaging maps favoring the non‐deprived eye (Espinosa and Stryker 2012; Gordon and Stryker 1996). Interestingly, the extent of ocular dominance plasticity in mice is dependent on strain, age, and environment, with enriched environments and dark exposure extending plasticity windows (Baroncelli et al. 2010; Sale et al. 2007). The relative contribution of callosal projections is more prominent in these species, and MD affects the balance of interhemispheric signaling, leading to cortical remapping even in the absence of ODCs (Restani and Caleo 2016).

One key distinction between these two groups lies in the presence and organization of binocular zones (Heesy 2004; Ibbotson and Jung 2020). In frontal‐eyed mammals, such as primates and cats, substantial portions of the primary V1 are dedicated to processing binocular input (Maier et al. 2022). This extensive binocular representation allows MD to induce shifts in the balance of eye‐specific inputs, leading to cortical reorganization (Espinosa and Stryker 2012). In contrast, the binocular zone in rodents is restricted to a narrow medial strip of V1 (~20% of its total width), which limits the spatial extent of MD‐driven reorganization (Mrsic‐Flogel et al. 2007). However, even within this narrow region, studies demonstrate synaptic weakening of deprived‐eye inputs and potentiation of open‐eye responses, mediated by N‐metil‐D‐aspartato (NMDA) receptor signaling, spine remodeling, and changes in inhibition (Maffei et al. 2006; Sawtell et al. 2003).

The cellular and molecular mechanisms of plasticity also diverge. In cats and monkeys, MD leads to loss of spine density, reduction in PV‐expressing interneurons, and degradation of PNNs, hallmarks of cortical disinhibition that permit synaptic remodeling (Hendry and Carder 1992; Nie and Wong‐Riley 1996). Similar phenomena are observed in rodents but are more temporally constrained (Beurdeley et al. 2012; Montey and Quinlan 2011; Pizzorusso et al. 2002). Neuromodulators such as BDNF, serotonin, and Neuregulin‐1 (NRG1)/ErbB4 appear conserved across species, although their expression gradients and target cell populations may vary (Gu et al. 2016; Wen et al. 2018). Notably, the presence of PNNs is species—and region‐dependent; while PNN degradation correlates with reopened plasticity in cats (Yin et al. 2006), its role in rodent lateral cortex is less pronounced (Carulli et al. 2010; Oray et al. 2004; Pizzorusso et al. 2006).

Functionally, frontal‐eyed species show deficits in high‐level visual behaviors post‐MD, including impaired binocular depth perception and motion integration (Harwerth et al. 1989; Kiorpes 2016; Movshon et al. 1987). MD affects extra‐striate areas, disrupting the normal development of receptive structure in V2 neurons, compromising spatial resolution, binocular, and orientation responses (Bi et al. 2011; Tao et al. 2014). These deficits are paralleled by long‐lasting cortical alterations even after binocular recovery, highlighting the vulnerability of binocular vision circuits (Chino et al. 1997). Lateral‐eyed species, lacking robust binocular vision, do not show such deficits (Prusky et al. 2000). However, MD in these animals still affects visual acuity and contrast sensitivity and is associated with glial activation and extracellular matrix remodeling in both V1 and associated areas (Murase et al. 2017, 2019).

The interpretation of MD effects must also be considered in ecological and evolutionary contexts (Nityananda and Read 2017). Frontal‐eyed predators, such as felids and primates, rely on stereopsis for hunting and object manipulation, placing evolutionary pressure on the development of precise binocular circuits (Parker 2007). In contrast, lateral‐eyed prey species prioritize panoramic vision for predator detection, which limits binocular integration but maximizes spatial field coverage (Heesy 2004). These divergent ecological demands have shaped distinct neural architectures and, consequently, different susceptibilities to amblyogenic insults.

Ultimately, the comparison between frontal‐ and lateral‐eyed mammals reveals both conserved and divergent themes in visual plasticity. While the presence of binocular circuitry magnifies the effects of MD in frontal‐eyed species, lateral‐eyed animals still manifest meaningful changes, particularly in glial responses, extracellular remodeling, and interhemispheric signaling. These findings argue for a broader definition of amblyopia‐related plasticity that encompasses species with non‐classical visual systems (Espinosa and Stryker 2012; Montey and Quinlan 2011; Murase et al. 2017).

A summary of these comparative mechanisms is provided in Table 1.

**TABLE 1:** Comparative mechanisms of visual cortical plasticity and glial responses following monocular deprivation in lateral‐ and frontal‐eyed mammals.

Mechanism/category	Frontal‐eyed species (cat and primate)	Lateral‐eyed species (mouse, rat, and rabbit)	Supporting evidence (references)
Ocular dominance columns (ODCs)	Present; reorganized following MD	Absent; diffuse, region‐specific reorganization	(Crair et al. 1998; LeVay et al. 1980; Mrsic‐Flogel et al. 2007)
Binocular zone extent in V1	Broad (~50%–70% of V1); highly susceptible to MD	Narrow (~20% of V1); limited to medial binocular strip	(Heesy 2004; Maier et al. 2022; Mrsic‐Flogel et al. 2007)
Parvalbumin interneuron response	Reduced density post‐MD; linked to cortical disinhibition	Also reduced but with less spatial and temporal extent	(Fagiolini and Hensch 2000; Hensch 2005a; Montey and Quinlan 2011)
Perineuronal nets (PNNs)	Degraded after MD; enable reopening of plasticity in adulthood	Less dense; modest role in restricting plasticity	(Carulli et al. 2010; Oray et al. 2004; Pizzorusso et al. 2002)
Synaptic plasticity (LTP/LTD)	Prominent in ODCs, orientation, and spatial domains	Localized in the binocular zone; dependent on NMDA receptors and spine remodeling	(Hofer et al. 2009; Maffei et al. 2006; Sawtell et al. 2003)
Callosal projections	Minimal involvement in plastic changes	Major role in cortical reorganization following MD	(Laing et al. 2015; Restani and Caleo 2016)
Glial activation (astrocytes, microglia)	Microglia prune inactive synapses; astrocytes modulate TNF‐*α* signaling	Significant activation post‐MD even in absence of ODCs	(A. S. Duffy and Eyo, 2025; K. R. Duffy, 2025; Murase et al. 2017; Sipe et al. 2016; Tremblay et al. 2010a, 2010b)
Neuromodulators (BDNF, NRG1, serotonin)	Strongly regulate critical period timing and expression	Conserved mechanisms; expression patterns vary regionally	(Gu et al. 2016; Huang et al. 1999; Wen et al. 2018)
Extrastriate cortical plasticity	V2 and associative areas show persistent visual impairments	Limited to V1 and subcortical circuits; stereopsis deficit is recovered by transcranial direct current stimulation	(Bi et al. 2011; Castaño‐Castaño, Feijoo‐Cuaresma, et al., 2019; Castaño‐Castaño, Martinez‐Navarrete, et al., 2019; Prusky et al. 2000; Tao et al. 2014)
Post‐MD visual behavior	Impaired stereopsis, depth perception, and motion integration	Decreased visual acuity and contrast sensitivity; stereopsis less affected	(Harwerth et al. 1989; Kiorpes 2016; Prusky et al. 2000)

## 4 Plasticity in Frontal‐ and Lateral‐Eyed Visual Cortical Systems

Cross‐species comparisons reveal that while the general mechanisms of cortical plasticity are conserved, their expression is tailored to the anatomical and ecological demands of the species. For example, in rodents, visual cortical plasticity relies more heavily on callosal connectivity and lateral interactions, given their relatively underdeveloped binocular integration (Espinosa and Stryker 2012; Levelt and Hübener 2012; Restani and Caleo 2016). In contrast, primates and carnivores exhibit more modular and columnar plastic changes, reflective of their need for precise binocular alignment and stereopsis. Additionally, species‐specific differences in the timing of critical periods and the density of PNNs influence both the onset and reversibility of MD‐induced changes (Berardi et al. 2000, 2004; Hensch 2005b; Hubel and Wiesel 1977; Kiorpes 2016; Shatz and Stryker 1978).

Frontal‐ and lateral‐eyed species differ in the timing and closure of critical periods and in the expression of molecular brakes on plasticity (e.g., perineuronal nets and myelin‐associated inhibitors). Understanding these differences translates into biomarker‐driven timing of interventions, for example, using imaging or cerebrospinal fluid assays to determine when and how pharmacological approaches reopen plasticity in adults with amblyopia (Carulli and Verhaagen 2021; Nabel and Morishita 2013; Shmal et al. 2024).

Cross‐species data reveal the extent to which genetic, molecular, and environmental factors shape cortical plasticity. Research in frontal‐ and lateral‐eyed species with different plasticity mechanisms suggests that multimodal therapies, for example, fluoxetine + perceptual training or environmental enrichment + noninvasive stimulation, may be necessary to reactivate dormant circuits. Understanding how these combinations work across species informs rational therapy design in humans (Maya‐Vetencourt and Origlia 2012; Saccenti et al. 2024; Voss et al. 2013).

The visual cortex of all species so far investigated, independent of the extent of binocular and monocular fields, shares a remarkable capacity for plasticity, particularly during the critical period of early postnatal development (Takahata 2024). During this phase, in both lateral‐ and frontal‐eyed species, sensory experiences profoundly shape synaptic organization, receptive field properties, and functional connectivity across the visual pathway (Espinosa and Stryker 2012; Hensch 2005a; Hubel and Wiesel 1970; Levelt and Hübener 2012). This plasticity underlies the capacity for recovery from visual impairments but also the vulnerability to long‐lasting deficits when inputs are imbalanced, such as in amblyopia (Kiorpes 2016; Mitchell and Maurer 2022). Although plasticity continues into adulthood, it becomes significantly constrained due to structural consolidation and the emergence of molecular “brakes” on circuit reorganization (Hensch 2005b; Pizzorusso et al. 2002).

Visual cortical plasticity can be described at multiple levels—from molecular signaling pathways and cellular architecture to circuit‐level modifications. At the cellular level, experience‐dependent changes involve synaptic strengthening or weakening (long‐term potentiation and depression), structural remodeling of dendritic spines, and dynamic shifts in inhibitory‐excitatory balance (Hofer et al. 2009; Keck et al. 2013; Toyoizumi et al. 2013). Activity‐dependent gene expression programs tightly regulate these modifications and involve multiple classes of neurons and glial cells. Notably, GABAergic interneurons, particularly those expressing PV, play a critical role in gating plasticity. The maturation of PV interneurons coincides with the opening of the critical period, and their modulation has been shown to extend or reinstate plasticity in adulthood (Donato et al. 2015; Hensch 2005b).

One of the defining features of critical period closure is the emergence of perineuronal nets (PNNs), specialized extracellular matrix structures that enwrap PV interneurons and restrict synaptic remodeling (Berardi et al. 2004; Carulli et al. 2010; Sorg et al. 2016; D. Wang and Fawcett 2012). The degradation of PNNs using chondroitinase ABC has been shown to restore plasticity in the adult visual cortex, emphasizing their role as molecular gatekeepers of structural stability (Kwok et al. 2011; Pizzorusso et al. 2002; Sorg et al. 2016). In addition to PNNs, epigenetic regulators, myelination, and neuromodulatory systems (e.g., BDNF, serotonin, and acetylcholine) collectively influence the timing and expression of plasticity (Di Cristo et al. 2001; Gu et al. 2016; Hensch and Bilimoria 2012; Putignano et al. 2007).

Plasticity in the visual cortex is also characterized by its layer‐ and region‐specific dynamics. Layer 4, the principal recipient of thalamic input, is highly responsive to changes in sensory drive, whereas Layers 2/3 participate in intracortical integration and exhibit longer‐term remodeling (Levelt and Hübener 2012; Medini 2011; Trachtenberg et al. 2000). In frontal‐eyed species, visual cortical maps are topographically organized into ODCs, orientation columns, and spatial frequency domains, all of which undergo experience‐dependent refinement. Disruption of visual input via MD leads to a reallocation of territory among ODCs, sharpening of orientation selectivity in the non‐deprived eye, and alterations in columnar width (Crair et al. 1998; Hubel and Wiesel 1977; LeVay et al. 1980). In contrast, lateral‐eyed species such as rodents lack prominent ODCs and instead display broader, overlapping monocular and binocular domains whose plasticity is less spatially segregated but nonetheless functionally significant (Dräger 1975; Gordon and Stryker 1996; Mrsic‐Flogel et al. 2007).

Astrocytes and microglia have emerged as critical regulators of cortical plasticity. These glial cells modulate synaptic pruning, secrete trophic factors, and participate in homeostatic scaling. Microglia, in particular, have been shown to engulf fewer active synapses during MD, facilitating ocular dominance shifts (Chung et al. 2015; Sipe et al. 2016; Tremblay et al. 2010). Astrocytic signaling via TNF‐*α* and other gliotransmitters contributes to synaptic remodeling and stabilization, especially in the context of activity deprivation (Beattie et al. 2002; Santello et al. 2019; Stellwagen and Malenka 2006). Thus, the plasticity of the visual cortex is not solely a neuronal phenomenon but a concerted process involving neuron–glia interactions, extracellular matrix dynamics, and transcriptional regulation.

From a translational standpoint, harnessing plasticity in the visual cortex holds promise for treating amblyopia beyond the traditional window of opportunity. Pharmacological and behavioral strategies aimed at reopening critical period‐like plasticity—such as fluoxetine, environmental enrichment, and visual perceptual training—have shown efficacy in animal models and early human studies (Bavelier et al. 2010; Levi 2020; Vetencourt et al. 2008; Polat et al. 2004; Sale et al. 2007).

A better understanding of frontal‐ and lateral‐eyed dependent plasticity may help inform which neural circuits are most plastic or targetable in individuals with varying visual profiles (e.g., central vs. peripheral visual field dominance and binocular alignment deficits) (Cang et al. 2023; Najafian et al. 2019; Takahata 2024).

These studies highlight the importance of a multimodal approach, integrating pharmacological, behavioral, and environmental strategies, to effectively harness neuroplasticity for therapeutic purposes.

## 5 Comparative Biology of the Critical Period

The concept of the critical period refers to a finite developmental window during which the nervous system is especially sensitive to experience and capable of undergoing profound plastic reorganization. In the visual cortex, this period is pivotal for the maturation of sensory circuits and for establishing functional binocularity (Hubel and Wiesel 1962; Mitchell and Maurer 2022). The closure of the critical period marks the transition to a more stable, less plastic cortical state and is associated with the emergence of molecular and structural constraints on synaptic remodeling (Benoit et al. 2015; Harauzov et al. 2010; Hensch and Bilimoria 2012; Levelt and Hübener 2012). Understanding the mechanisms that regulate the onset, progression, and closure of this period is crucial for designing interventions that aim to recover visual function in amblyopia and other developmental disorders (Hensch and Quinlan 2018).

From a developmental perspective, the temporal profile of the critical period varies across species and cortical areas. In mice, the critical period for ocular dominance plasticity peaks around postnatal Day 28 (P28) and closes by P35–P40, whereas in cats and primates it extends over a more prolonged timeline (Antonini et al. 1999; Espinosa and Stryker 2012; Fagiolini et al. 1994; Takahata 2024; J. Wang et al. 2019). These interspecies differences reflect divergent patterns of cortical maturation and are likely shaped by ecological demands (Nityananda and Read 2017). In frontal‐eyed species with complex visual ecologies, the extended critical period permits fine‐tuning of binocular integration and depth perception (Mitchell and Maurer 2022). In contrast, lateral‐eyed species may experience a more temporally restricted plastic window, aligned with their reliance on monocular cues and broader visual field coverage (Laing et al. 2015; Takahata 2024).

The initiation of the critical period in the primary visual cortex (V1) independent of the extent of the binocular field is tightly regulated by the maturation of inhibitory circuitry, particularly fast‐spiking, PV‐expressing GABAergic interneurons (Di Cristo et al. 2007; Lazarus and Huang 2011). This relationship has been extensively documented in frontal‐eyed species, such as cats and primates, where the development of these inhibitory neurons sets the timing for heightened cortical plasticity during early postnatal life (Hensch 2005a). In lateral‐eyed species, like rodents, similar mechanisms have been observed. For instance, studies in mice have demonstrated that the maturation of PV+ interneurons is crucial for initiating critical period plasticity in the visual cortex (Fagiolini et al. 2004; Fagiolini and Hensch 2000). Experimental studies in mice have shown that pharmacological enhancement of GABAergic transmission with benzodiazepines can trigger premature onset of the critical period, whereas GABAergic blockade delays its initiation (Hensch 2005a; van Versendaal and Levelt 2016). PV+ interneurons shape the excitatory–inhibitory balance within local circuits, and their perisomatic synapses play a critical role in modulating cortical responsiveness. Their maturation is regulated by both intrinsic genetic programs and extrinsic sensory experience (Baho et al. 2019; Chattopadhyaya 2011; Rupert and Shea 2022).

Once initiated, the critical period is influenced by a network of cellular and molecular factors that determine its duration and closure. A major contributor is the formation of PNNs—specialized extracellular matrix aggregates that preferentially enwrap PV+ interneurons and restrict structural plasticity (Carulli et al. 2010; Carulli and Verhaagen 2021; Grødem et al. 2025; Wingert and Sorg 2021). The degradation of PNNs using chondroitinase ABC has been shown to reopen plasticity in the adult visual cortex, indicating their pivotal role as molecular “brakes” (Lesnikova et al. 2021; Pizzorusso et al. 2006). The timing of PNN maturation is closely aligned with the decline in ocular dominance plasticity, suggesting a causal relationship (Lensjø et al. 2017; Sigal et al. 2019). Other structural elements, such as myelin‐associated proteins (e.g., Nogo‐A and MAG), also contribute to limit synaptic plasticity and stabilize circuit architecture (McGee et al. 2005; Mdzomba et al. 2018; Pernet 2017; Stewart et al. 2022).

Neuromodulatory systems are essential regulators of critical period dynamics. BDNF, for instance, is both necessary and sufficient to initiate the critical period in rodents (Gianfranceschi et al. 2003; Huang et al. 1999; Wong‐Riley 2021; Zhang et al. 2018). Its expression is activity‐dependent and exerts trophic effects on both excitatory and inhibitory neurons. Serotonergic and cholinergic systems further modulate cortical excitability and influence synaptic plasticity and manipulations that increase neuromodulatory tone. Indeed, environmental enrichment or pharmacological agents have been shown to prolong or reinstate plasticity (Castrén and Antila 2017; Higa et al. 2024; Vetencourt et al. 2008; Sale et al. 2007; Stryker and Löwel 2018).

Recent studies have identified molecular markers that delineate the boundaries of the critical period. The transcription factor Otx2, secreted by the choroid plexus and internalized by the PV+ interneurons, has been shown to control the timing of PNN formation and the onset of plasticity (Beurdeley et al. 2012; Sakai and Sugiyama 2018; Sugiyama et al. 2008). Blocking Otx2 uptake prevents PNN maturation and sustains a juvenile‐like state of plasticity in the visual cortex (Carulli and Verhaagen 2021). Similarly, the homeobox gene MeCP2, which regulates chromatin architecture, modulates critical period timing, and mutations in this gene are associated with neurodevelopmental disorders including Rett syndrome (Krishnan et al. 2015). These findings underscore the importance of epigenetic and extracellular signaling in the orchestration of developmental plasticity (Picard and Fagiolini 2019; Sakai and Sugiyama 2018).

Glial cells, including astrocytes and microglia, are increasingly recognized as active participants in the regulation of the critical period. Astrocytes release gliotransmitters and extracellular matrix components that influence synaptic stability and pruning (Allen and Eroglu 2017; Christopherson et al. 2005; Vivi and Di Benedetto 2024). Microglia, through phagocytic and cytokine‐mediated mechanisms, help to eliminate weak or unnecessary synapses and shape circuit architecture in response to sensory experience (Cornell et al. 2022; Schafer et al. 2012; Tremblay et al. 2010). Importantly, glial maturation is itself experience‐dependent, indicating bidirectional interactions between neural activity and glial development (Starkey et al. 2023).

Experimental interventions aimed at manipulating the critical period have generated promising strategies for amblyopia therapy. Environmental enrichment, characterized by increased sensory, cognitive, and motor stimulation, reactivates plasticity and facilitates functional recovery in adult animals (Baroncelli et al. 2010; Sale et al. 2007; Shmal et al. 2024; Tognini et al. 2012). Pharmacological treatments such as fluoxetine, valproate, and histone deacetylase inhibitors have been employed to modulate epigenetic states and restore synaptic malleability (Vetencourt et al. 2008; Maya‐Vetencourt and Origlia 2012; Putignano et al. 2007). Non‐invasive stimulation methods, including repetitive transcranial magnetic stimulation and binocular visual training (Liu et al. 2024; Tsaousis et al. 2023; Zheng et al. 2023), are currently under investigation in humans with promising preliminary results (Tuna et al. 2020, 2024).

Critical period regulation is thus a multifactorial process, integrating genetic, molecular, structural, and environmental inputs. By elucidating the interplay of these factors across species, we can better understand how amblyopia arises and why its reversibility varies between individuals and species. Moreover, identifying shared and divergent regulatory mechanisms in frontal‐ and lateral‐eyed species offers a roadmap for tailoring interventions to specific developmental windows and neuroanatomical contexts.

Ultimately, the comparative biology of the critical period serves as a cornerstone for designing evidence‐based, neuroplasticity‐enhancing therapies that extend the window of opportunity for functional recovery (Birch and Duffy 2024; Hensch and Quinlan 2018; Thompson et al. 2024).

## 6 Conclusions and Future Directions

Amblyopia remains a complex and multifaceted neurodevelopmental disorder whose study continues to provide critical insights into the principles of brain plasticity. As demonstrated throughout this review, the comparative analysis of MD effects across frontal‐ and lateral‐eyed mammals not only elucidates the shared and divergent mechanisms underlying visual cortical plasticity but also highlights the importance of tailoring experimental models to reflect species‐specific visual ecologies. Frontal‐eyed species, such as cats and primates, with their extensive binocular overlap and ODC organization, offer robust systems for tracking deprivation‐induced shifts in cortical territory. In contrast, lateral‐eyed species, such as rodents, offer complementary insights into plasticity mechanisms driven more by interhemispheric communication, subcortical inputs, and glial modulation.

Key findings from lateral‐eyed rodent models—especially those incorporating genetic tools, longitudinal imaging, and behavioral assays—have provided a granular understanding of how critical periods are regulated, how plasticity is gated by perineuronal nets and glial interactions, and how environmental and pharmacological interventions can reopen plasticity windows. Comparative research on frontal—and lateral‐eyed species plasticity emphasizes a fundamental principle: structure shapes function, and both differ across individuals. By drawing on insights from diverse animal models, we can better predict how individuals will respond to treatment, tailor therapies to target the most relevant neural circuits, and design interventions appropriate for a person's age and anatomical profile. This approach represents a shift from generalized treatment strategies toward precision rehabilitation, mirroring the advances in precision medicine.

Despite these gains, several challenges remain. Translational gaps persist between rodent models and human applications, particularly in the realm of behavioral generalization and long‐term efficacy. Furthermore, variability in treatment outcomes underscores the need for better biomarkers to predict responsiveness and guide therapy. Ethical and logistical concerns also arise in the clinical deployment of emerging technologies such as gene editing, intracortical stimulation, or sustained‐release neuroactive compounds.

However, comparative studies across species with differing visual system architectures will remain essential, not only for validating universal principles of plasticity but also for identifying targets unique to specific anatomical configurations. By recognizing that distinct structural features—such as binocular field overlap, ODC presence, and glial response profiles—profoundly shape the plastic potential of each system, we pave the way for personalized therapeutic strategies in amblyopia. This cross‐species framework not only enriches our understanding of neuroplasticity but also bridges the translational gap, bringing us closer to precision medicine approaches for visual rehabilitation.

## Author Contributions

**Fábio Leite do Amaral Júnior:** conceptualization, data curation, investigation, methodology, visualization. **Thalyta Alves Rodrigues:** conceptualization, data curation, investigation, methodology. **Nonata Lúcia Trévia da Silva:** conceptualization, data curation, investigation, methodology. **Izabela Negrão Almeida Diniz:** conceptualization, data curation, formal analysis, visualization. **Luciana Negrão Almeida de Morais:** conceptualization, data curation, formal analysis, visualization. **Daniel Guerreiro Diniz:** conceptualization, data curation, formal analysis, visualization. **Dora Brites:** formal analysis, supervision, validation, writing – original draft, writing – review and editing. **Daniel Clive Anthony:** formal analysis, supervision, validation, writing – original draft, writing – review and editing. **Cristovam Wanderley Picanço Diniz:** formal analysis, supervision, validation, writing – original draft, writing – review and editing.

## Conflicts of Interest

The authors declare no conflicts of interest.

## Peer Review

The peer review history for this article is available at https://www.webofscience.com/api/gateway/wos/peer‐review/10.1111/ejn.70179.

## Data Availability

The datasets generated and/or analyzed during the current study are available from the corresponding author upon reasonable request.
